# Alanine Scanning Studies of the Antimicrobial Peptide Aurein 1.2

**DOI:** 10.1007/s12602-018-9501-0

**Published:** 2018-12-19

**Authors:** Dorian Migoń, Maciej Jaśkiewicz, Damian Neubauer, Marta Bauer, Emilia Sikorska, Elżbieta Kamysz, Wojciech Kamysz

**Affiliations:** 1 Department of Inorganic Chemistry, Faculty of Pharmacy, Medical University of Gdańsk, Al. Gen. J. Hallera 107, 80-416 Gdańsk, Poland; 2 Polpharma Biologics, Gdańsk, Poland; 3 Faculty of Chemistry, University of Gdańsk, Gdańsk, Poland

**Keywords:** Aurein 1.2, Antimicrobial peptides, Structure-activity relationship, Antimicrobial agents, Peptide drugs, Antibiotics

## Abstract

Antimicrobial peptides (AMPs) are compounds widely distributed in nature that display activity against a broad spectrum of pathogens. Amphibian skin, as an organ rich in pharmacologically active peptides, appears to be an interesting source of novel AMPs. Aurein 1.2 (GLFDIIKKIAESF-NH_2_) is a short 13-residue antimicrobial peptide primarily isolated from the skin secretions of Australian bell frogs. In this study, the alanine scan of aurein 1.2 was performed to investigate the effect of each amino acid residue on its biological and physico-chemical properties. The biological studies included determination of minimum inhibitory concentration, activity against biofilm, and inhibitory effect on its formation. Moreover, the hemolytic activity as well as serum stability was determined. The hydrophobicity of peptides and their self-assembly were investigated using reversed-phase chromatography. In addition, their helicity was calculated from circular dichroism spectra. The results not only provided information on structure-activity relationship of aurein 1.2 but also gave insights into design of novel analogs of AMPs in the future.

**Electronic supplementary material:**

The online version of this article (10.1007/s12602-018-9501-0) contains supplementary material, which is available to authorized users.

## Introduction

An increasing resistance to antibiotics among microorganisms looms a serious threat to human society. According to the Review on Antimicrobial Resistance, at least 700,000 annual deaths are caused by drug-resistant infections. Furthermore, this number was estimated to soar up to 10 million in 2050, if only the current antibiotic politics remain unchanged [1]. There are several reasons of this situation and due, among others, to inappropriate antibiotic prescribing, extensive agricultural use, and limited access to new antibiotics [2]. In this situation, the development of antimicrobial agents with improved modes of action and low-resistance potential is needed. Antimicrobial peptides (AMPs) as a class of compounds meeting those requirements offer an interesting alternative to conventional antibiotics. AMPs are endogenic molecules widely distributed in nature, mainly as a part of innate immune system. Generally, they are 11–50 amino acid residue long, amphipathic molecules with net positive charge at neutral pH [3]. The main mechanism of their antimicrobial activity is based on a non-receptor-mediated microbial membrane disruption. Moreover, AMPs lead to microbial cell death via other mechanisms such as inhibition of protein/DNA or cell wall synthesis or induction of apoptosis/necrosis. Owing to those properties, AMPs display antimicrobial activity against a broad spectrum of pathogens such as Gram-positive and Gram-negative bacteria, fungi, protozoa, and viruses. Because of the membrane-associated activity, they could also exhibit a low potency to trigger antimicrobial resistance [4]. Amphibian skin, as an organ rich in pharmacologically active peptides, appears to be an interesting source of novel AMPs. Physiologically, it is responsible for regulation of biological fluids equilibrium, respiration, and transportation of essential ions. To assure efficient and unlimited course of these processes, amphibian skin is adequately thin and humid. This in turn increases the risk of infection. Because of that, amphibians had evolutionarily developed a series of defense mechanisms, with AMP biosynthesis being one of them [5, 6]. Until now, about 1000 amphibian AMPs have been isolated and examined [7]. However, because of species multiplicity, their total number could reach even 100,000 individual compounds [8]. Unfortunately, AMPs are usually chemically and biologically unstable, and relatively toxic towards human cells. However, chemical synthesis of analogs based on those AMPs can lead to more stable and active candidates for antimicrobials [9]. One of the tools used for a better understanding of peptide activity is alanine scanning. This approach allows to determine the contribution of each amino acid residue to specific peptide features what helps to rationalize further modifications. Aurein 1.2 (GLFDIIKKIAESF-NH_2_) is a short 13-residue antimicrobial peptide with molecular mass of approximately 1480 Da and positive (+ 1) net charge at physiological pH. It was initially isolated from Australian bell frogs, *Ranoidea aurea* (formerly *Litoria aurea*) and *Ranoidea raniformis* (formerly *L. raniformis*) skin secretions [10]. Antimicrobial activity of aurein 1.2 ranges between 1 and 16 μg/mL in terms of minimum inhibitory concentrations (MICs) against both reference and clinical strains of Gram-positive bacteria such as *Staphylococcus aureus*, *Enterococcus faecalis*, or *Streptococcus pyogenes* (including methicillin-resistant *S. aureus* and vancomycin-resistant *E. faecalis*). Moreover, a synergistic effect between aurein 1.2 and minocycline/clarithromycin against those bacteria has also been reported [11]. On the other hand, in vitro studies demonstrated aurein 1.2 to exhibit a low antimicrobial activity against *Escherichia coli* and *Pseudomonas aeruginosa* (MIC values for both strains are 256 μg/mL) [12] and moderate activity against *Candida albicans* strains (MIC value of 32 μg/mL) [13]. Additionally, anticancer activity of aurein 1.2 has been demonstrated through the NCI-60 human tumor cell lines [14]. It exerts cytotoxic activity against several human neoplastic cell lines of leukemia and melanoma as well as the lung, colon, central nervous system ovarian, prostate, and breast cancer cell lines [10]. Aurein 1.2 does not adopt any secondary structure in aqueous media. After incorporation into the lipid membrane, it adopts an α-helical geometry. Fernandez. et al. demonstrated that aurein 1.2 exerts disruptive activity via carpet-like mechanism against both neutral and anionic model membranes, with a greater affinity towards the latter [15]. Furthermore, significant alternation of lipid components between the negatively charged model membrane leaflets due to aurein 1.2 binding has also been demonstrated [16]. Laadhari et al. in their in vivo studies suggest that antimicrobial activity of aurein 1.2 is not only an effect of membrane destabilization and/or disruption, but also interactions between the peptide and bacterial teichoic acid and lipoteichoic acid should be considered [17].

In this study, alanine scanning of aurein 1.2 was performed to recognize the effect of each amino acid residue on its biological and physico-chemical properties. For this purpose, multiple parameters were measured, including the following: hydrophobicity, helicity, biological activity, serum stability, and the tendency to self-association. The results not only provided the information on the structure-activity relationship of aurein 1.2 but also gave insights into design of novel analogs of AMPs in the future.

## Materials and Methods

### Peptide Synthesis

Aurein 1.2 and its analogs were synthesized manually according to the standard *N*^*α*^-Fmoc protocol on a Rink Amide resin (0.7 mmol/g substitution). Peptide chains were elongated using repeated cycles of deprotection and acylation. Deprotection of Fmoc groups was conducted using a 20% piperidine solution in DMF (1 × 20 min). Coupling reactions were carried out using 3 eq. of protected amino acids (Fmoc-AA), Oxyma Pure, and DIC (1:1:1) dissolved in DCM/DMF. Peptide cleavage and side-chain deprotection were accomplished in 1 h through a one-step procedure using a mixture of TFA/TIS/H_2_O (95:2.5:2.5). The crude peptides were precipitated with cold anhydrous diethyl ether and lyophilized. Subsequently, the peptides were purified by reversed-phase high-performance liquid chromatography (RP-HPLC) with LP-chrom software. Purifications were carried out on a Phenomenex Gemini-NX C18 column (21.20 × 100 mm, 5-μm particle size, 110-Å pore size). UV detection at 214 nm was used, and the crude peptides were eluted with a linear 10–60% acetonitrile gradient in deionized water over 60 min at room temperature. The mobile phase flow rate was 10.0 mL/min. Both eluents contained 0.1% (*v*/*v*) of TFA. Fractions were analyzed on a Waters XBridge Shield RP-18 column (4.6 × 150 mm, 3.5-μm particle size, 130-Å pore size) with UV detection at 214 nm. Pure fractions (> 95%, by HPLC analysis) were collected and lyophilized. The molecular weights were confirmed by ESI-MS.

### Antimicrobial Activity

The minimum inhibitory concentrations (MICs) of test compounds were assessed by broth microdilution method on 96-well plates, according to the guidelines of Clinical and Laboratory Standards Institute (CLSI) [18, 19]. For this purpose, reference strains of bacteria: *S. aureus* ATCC 25923, *E. faecalis* PCM 2673, *E. coli* ATCC 25922, and *P. aeruginosa* ATCC 9027; and fungus: *C. albicans* ATCC 10231 were used (The Polish Collection of Microorganism, Polish Academy of Sciences, Wrocław, Poland). Bacteria at initial inoculums of 0.5 × 10^5^ CFU/mL in Mueller Hinton Broth (MHB), and fungi at initial inoculums of 2 × 10^3^ CFU/mL in RPMI-1640, were exposed to the compounds over the concentration range of 1–512 μg/mL. The polystyrene plates were incubated at 37 °C for 18 h with bacteria and for 24 h with *C. albicans*. The MIC was taken as the lowest concentration at which a noticeable growth of microorganisms was inhibited. Minimum biofilm eradication concentrations (MBECs) were determined using a previously described protocol [20]. For this purpose, 96-well polystyrene flat-bottom plates and a resazurin (7-hydroxy-3H-phenoxazin-3-one 10-oxide) were used. In this assay, a specific feature of resazurin is utilized, which upon the contact with living cells is metabolized and reduced from the basic blue form to pink resorufin. Briefly, 24-h cultures of microorganisms were diluted 1,000-fold and 100 μL of that suspension was added into the test plates. After 24 h of incubation at 37 °C, the wells of the plates were rinsed three times with phosphate buffer saline (PBS) to remove non-adherent cells. Subsequently, 100 μL of test compounds in concentration range (diluted in appropriate media) were added to each well. After 24 h of incubation at 37 °C, 20 μL of the resazurin (4 mg/mL) was added. The MBEC was read after 1 h. Additionally, anti-adhesive properties of the compounds were also analyzed. The assay was conducted on 96-well polystyrene flat-bottom plates. The preparation of inoculums was followed by a 500-fold dilution of 24-h cultures of microorganisms. Briefly, to evaluate the effect of the peptides in preventing biofilm formation, 50 μL of compounds in concentration range, diluted in appropriate medium, were prepared. Subsequently, the 50 μL of the bacterial/fungal inoculums were added to reach the same microbial density as that in MBEC assay. After 24 h of incubation at 37 °C, the wells were rinsed three times with PBS and the fresh media with resazurin (0.6 mg/mL) were added. Then, after 1 h of incubation, the minimum biofilm inhibitory concentrations (MBIC) were read. MBECs as well as MBICs were determined as the lowest concentration at which the reduction of resazurin was lower or equal (10% ± 0.5%) as compared to positive (100%) and negative (0%) controls. All experiments were performed in triplicate.

### Hemolysis Assay

The hemolytic activity of the peptides was determined using fresh human red blood cells (RBCs) collected from a healthy human donor according to the procedure described in the literature [21]. Fresh RBCs with EDTA as anticoagulant were rinsed three times with a PBS by centrifugation at 800×*g* for 10 min and resuspension in PBS. Serial dilution of the peptides in PBS (1–256 μg/mL) on 96-well polystyrene plates was prepared and the stock solution of RBCs was added up to a final volume of 100 μL with a 4% concentration of erythrocytes (*v*/*v*). The control wells for 0 and 100% hemolysis contained of RBCs suspended in PBS and 1% of Triton-X 100, respectively. Subsequently, the plates were incubated for 1 h at 37 °C and then centrifuged at 800×*g* for 10 min at 4 °C (Sorvall ST 16R Centrifuge, Thermo Scientific). After centrifugation, the supernatant was carefully resuspended to new microtiter plates and the release of hemoglobin was measured at 540 nm (Multiskan™ GO Microplate Spectrophotometer, Thermo Scientific). All experiments were conducted in triplicate.

### Determination of Peptide Hydrophobicity Parameter

The hydrophobicity parameter was expressed as RP-HPLC mobile phase acetonitrile content (%ACN) at a retention time of a particular peptide. All peptides were analyzed using Waters Alliance e2695 system with a Waters 2998 PDA Detector (software–Empower®3). All HPLC analyses were conducted in triplicate on a Waters XBridge™ Shield RP-18 column (4.6 × 150 mm, 3.5-μm particle size, 130-Å pore size) using a linear 30–60% ACN (B) gradient in deionized water (A) over 30 min (1% ACN/min) at 25.0 ± 0.1 °C, at a flow rate of 0.5 mL/min and UV detection at 214 nm (A–0.1% aqueous TFA and B–0.1% TFA in ACN).

### Determination of Peptide Self-Association

Peptide molecules exist in solution as monomeric and/or self-associated species. In most cases, peptide self-association occurs through hydrophobic interactions between their non-polar faces. Interestingly, in order to interact with hydrophobic stationary phase, peptide molecule needs to be in its monomeric form (i.e., needs to have its non-polar face unoccupied). Because of this, self-associated species disruption, being a result of temperature increase, result in longer retention time of peptide. On the other hand, increasing temperature causes also a decrease in mobile phase viscosity and increase in mass transfer between the mobile and stationary phase. These, in turn, contribute to accelerated retention and stand in contrast to the effect caused by disruption of self-associates. Peptide tendency to self-association may be characterized by a maximum (*T*_max_) on a normalized plot of the change in retention time at different temperatures. At *T*_max_, the impact of these different effects on retention time is equal [22]. Self-association of the peptides was determined using RP-HPLC temperature profiling analyses. All peptides were analyzed using Waters Alliance e2695 system with a Waters 2998 PDA Detector (software–Empower®3). The temperature ranged from 5 to 60 °C at 5 °C increments (± 0.1 °C). Analyses were performed in triplicate at each temperature using conditions described in detail in the preceding section. Peptide retention times were normalized by subtraction with the retention time value obtained at 5 °C and plotted over adequate temperature values. Peptide self-association tendency was expressed as *T*_max_.

### Serum Stability

To assess serum stability, the peptides (0.5 mg) were dissolved in 4 mL of 25% (*v*/*v*) fetal bovine serum (FBS) in PBS and incubated at 37 °C. The aliquots of 100 μL of the test samples were transferred to 96-well polystyrene plates in triplicate for each time point. Samples were quenched at zero time (*t*_0_) and after 6 h of incubation at 37 °C by addition of 100 μL of ice-cold acetonitrile and stored at 4 °C for 10 min. Afterwards, the samples were centrifuged at 13,000×*g* for 20 min at 4 °C and diluted 1:1 with HPLC-grade water. All samples were analyzed using the Waters Alliance e2695 system with Waters Acquity QDa Detector (software–Empower®3). Analyses were conducted on an Agilent Zorbax SB-C18 RP-18 column (4.6 × 50 mm, 1.8-μm particle size, 80-Å pore size) using a linear 30–60% acetonitrile (B) gradient in deionized water (A) over 6 min at 25.0 ± 0.1 °C, at a flow rate of 0.5 mL/min and MS working in selected ion recording (SIR) mode (A—0.1% aqueous formic acid (FA) and B—0.1% FA in acetonitrile). The percentage of peptide remaining intact was calculated from peak areas of samples at t_0_ and after 6 h of incubation in serum (the % of remaining peptide = peak area after 6 h/peak area at *t*_0_). The mass-to-charge ratios selected for SIR mode were based on values obtained from full spectrum analysis of each peptide and are presented in Table 1.

**Table 1:** Peptides synthesized in this study

Peptide	Sequence	Average mass [Da]	Net charge	MS analysis		
				*z* ^a^	*m*/*z*^b^	*m*/*z*^c^
G1A	**A**LFDIIKKIAESF-NH_2_	1493.79	+ 1	3 2	498.93 747.90	498.98 *747.90*
L2A	G**A**FDIIKKIAESF-NH_2_	1437.68	+ 1	3 2	480.23 719.84	480.31 *719.64*
F3A	GL**A**DIIKKIAESF-NH_2_	1403.67	+ 1	3 2	468.89 702.84	468.95 *702.63*
D4A	GLF**A**IIKKIAESF-NH_2_	1435.75	+ 2	3 2	479.58 718.88	479.58 *718.79*
I5A	GLFD**A**IKKIAESF-NH_2_	1437.68	+ 1	3 2	480.23 719.84	480.36 *719.69*
I6A	GLFDI**A**KKIAESF-NH_2_	1437.68	+ 1	3 2	480.23 719.84	480.34 *719.76*
K7A	GLFDII**A**KIAESF-NH_2_	1422.67	0	2	712.34	*712.31*
K8A	GLFDIIK**A**IAESF-NH_2_	1422.67	0	2	712.34	*712.15*
I9A	GLFDIIKK**A**AESF-NH_2_	1437.68	+ 1	3 2	480.23 719.84	480.36 *719.84*
E11A	GLFDIIKKIA**A**SF-NH_2_	1421.72	+ 2	3 2	474.91 711.86	474.92 *711.57*
S12A	GLFDIIKKIAE**A**F-NH_2_	1463.76	+ 1	3 2	488.92 732.88	488.90 *732.72*
F13A	GLFDIIKKIAES**A**-NH_2_	1403.67	+ 1	3 2	468.89 702.84	468.91 *702.69*
aurein 1.2	GLFDIIKKIAESF-NH_2_	1479.76	+ 1	3 2	494.25 740.88	494.32 *740.74*

^a^Ion charge

^b^Calculated mass-to-charge ratio;

^c^Measured mass-to-charge ratio, italicized values are mass-to-charge ratios selected for SIR LC-MS analysis

### CD Measurements

The CD spectra of aurein 1.2 and its alanine scanning analogs were recorded on a JASCO J-815 spectropolarimeter at 25 °C over the 190–260-nm range under three different conditions, i.e., surfactant-free phosphate buffer at pH 7.4, buffered 20 mM SDS (sodium dodecyl sulfate), and 20 mM DPC (dodecylphosphocholine) micellar solutions. The peptide concentration was 0.15 mg/mL. Every spectrum was scanned three times to amplify the signal-to-noise ratio. The spectra were plotted against the mean residual molar ellipticity (MRME, deg. × cm^2^ × dmol^−1^) versus wavelength (nm). Deconvolution of the CD spectra was carried out using CDPro software with CONTINILL algorithm and SMP56 database set [23].

## Results

### Peptide Synthesis

Sequences, average masses, net charges, and calculated and measured mass-to-charge ratios of the synthesized peptides are shown in Table 1.

### Antimicrobial Assay

Among the test compounds, only three aurein 1.2 analogs exhibited satisfactory antimicrobial activity (Table 2). The most effective was D4A with a 3-fold lower MIC values against *E. coli* and *S. aureus*, and a 2-fold against *P. aeruginosa* than the original compound. With *E. faecalis*, it exhibited only the order of magnitude higher activity, while with *C. albicans* the opposite relation was found. In this case, D4A exhibited the order of magnitude lower activity. Another compound was E11A, with the MIC values from 1- to 2-fold dilutions lower against all bacterial strains and the same for *C. albicans*. For S12A, the higher activity was found only for *S. aureus* and the same for other strains with an exception for *P. aeruginosa*. For this strain, the growth was not inhibited at a whole concentration range. The remaining compounds were either ineffective or their MIC values were higher than or equal to 128 μg/mL with an exception for K8A and *C. albicans*. For this compound, the MIC value was a magnitude higher than for aurein 1.2. The MBEC values indicate that only D4A eradicated biofilm of all the test strains at concentrations used in the study (Table 3). The highest activity was found against *S. aureus* with MBEC value of 16 μg/mL, which was 4-fold lower than that of aurein 1.2. Identical result against *S. aureus* biofilm was found for E11A. Moreover, both compounds eradicated biofilm formed by *E. coli* at 32 and 64 μg/mL and *P. aeruginosa* at 512 μg/mL, respectively. It is noteworthy that analog S12A was as active as parent molecule against *C. albicans* biofilm, while other analogs exhibited higher MBEC. Furthermore, D4A and S12A were the only two compounds active against the *E. faecalis* biofilm (128 μg/mL). Interestingly, all the test compounds exhibited high anti-adhesive properties against *C. albicans* (Table 4); but, the highest activity was found for aurein 1.2, D4A, and S12A (2 μg/mL). D4A and E11A displayed high anti-adhesive properties for all the test bacterial strains, and these analogs were even more potent than aurein 1.2. Despite the fact that S12A was more effective for *S. aureus* and *E. coli*, it did not affect the biofilm formation of *P. aeruginosa*.

**Table 2:** The MIC (μg/mL) values of the test compounds against reference strains of microorganisms. MIC values of the analogs lower than those of aurein 1.2 are italicized

MIC [μg/mL]					
Peptide	*S. aureus* ATCC 25923	*E. faecalis* PCM 2673	*E. coli* ATCC 25922	*P. aeruginosa* ATCC 9027	*C. albicans* ATCC 10231
G1A	512	128	128	512	128
L2A	> 512	512	256	512	256
F3A	512	256	128	512	128
D4A	*16*	*32*	*8*	*64*	64
I5A	> 512	128	256	512	256
I6A	> 512	256	512	> 512	256
K7A	> 512	512	> 512	> 512	> 512
K8A	> 512	64	128	> 512	64
I9A	> 512	128	512	> 512	512
E11A	*32*	*32*	*16*	*64*	32
S12A	*32*	64	64	> 512	32
F13A	> 512	128	128	> 512	512
urein 1.2	128	64	64	256	32

**Table 3:** The MBEC (μg/mL) values of the test compounds against reference strains of microorganisms. MBEC values of the analogs lower than those of aurein 1.2 are italicized

MBEC [μg/mL]					
Peptide	*S. aureus* ATCC 25923	*E. faecalis* PCM 2673	*E. coli* ATCC 25922	*P. aeruginosa* ATCC 9027	*C. albicans* ATCC 10231
G1A	512	> 512	256	512	> 512
L2A	> 512	> 512	512	> 512	> 512
F3A	> 512	> 512	256	> 512	> 512
D4A	*16*	*128*	*32*	512	512
I5A	> 512	> 512	512	> 512	512
I6A	> 512	> 512	> 512	> 512	> 512
K7A	> 512	> 512	> 512	> 512	> 512
K8A	> 512	> 512	> 512	> 512	> 512
I9A	> 512	> 512	> 512	> 512	> 512
E11A	*16*	> 512	64	512	> 512
S12A	> 512	*128*	> 512	> 512	256
F13A	128	> 512	512	> 512	> 512
aurein 1.2	128	> 512	64	512	256

**Table 4:** The MBIC (μg/mL) values of the test compounds against reference strains of microorganisms. MBIC values of the analogs lower than that of aurein 1.2 are italicized

MBIC [μg/mL]					
Peptide	*S. aureus* ATCC 25923	*E. faecalis* PCM 2673	*E. coli* ATCC 25922	*P. aeruginosa* ATCC 9027	*C. albicans* ATCC 10231
G1A	512	256	128	256	4
L2A	> 512	> 512	512	> 512	8
F3A	>512	256	256	512	4
D4A	*16*	*32*	*8*	*64*	2
I5A	> 512	512	512	> 512	4
I6A	> 512	> 512	512	> 512	4
K7A	> 512	> 512	64	> 512	4
K8A	> 512	128	32	> 512	4
I9A	> 512	> 512	>512	> 512	16
E11A	*32*	*32*	*16*	*64*	4
S12A	*32*	128	*32*	>512	2
F13A	>512	>512	>512	>512	8
aurein 1.2	128	128	128	128	2

### Hemolysis Assay

Almost all the analogs were less hemolytically active against human RBCs than aurein 1.2. Only K8A was the compound with a right-shifted concentration curve, which means that at almost all concentrations the hemolytic properties were higher than that of the original compound. Among others, L2A, I6A, I9A, and F13A did not cause the lysis of RBCs over the at whole concentration range, while S12A was more hemolytic between 64 and 16 μg/mL (Table 5).

**Table 5:** Percentage of hemolysis [%] caused by test compounds at different concentrations [μg/mL]

Peptide	256	128	64	32	16	8	4	2	1
G1A	4.2	0.3	0.0	0.0	0.0	0.0	0.0	0.0	0.0
L2A	0.0	0.0	0.0	0.0	0.0	0.2	0.4	0.6	0.3
F3A	0.3	0.0	0.0	0.0	0.0	0.0	0.0	0.1	0.1
D4A	61.0	23.2	6.6	1.5	0.3	0.3	0.3	0.3	0.2
I5A	1.1	0.0	0.1	0.0	0.0	0.0	0.2	0.3	0.2
I6A	0.1	0.1	0.1	0.0	0.1	0.3	0.5	0.6	0.4
K7A	4.6	3.0	0.7	3.4	0.1	0.0	0.0	0.1	0.2
K8A	89.6	49.3	16.1	5.5	0.0	0.0	0.0	0.0	0.1
I9A	0.0	0.0	0.0	0.0	0.0	0.1	0.3	0.6	0.6
E11A	40.2	11.9	2.9	0.4	0.1	0.1	0.2	17.4	0.1
S12A	23.5	15.4	12.0	6.5	1.2	0.0	0.0	0.0	0.0
F13A	0.0	0.0	0.0	0.0	0,0	0.4	0.5	0.6	0.3
aurein 1.2	58.8	19.2	4.5	0.5	0.4	0.0	0.0	0.1	0.0

### Hydrophobicity Parameter of Peptides

Hydrophobicity parameter of a specific peptide was expressed as the percentage of acetonitrile in the mobile phase at the time of peptide elution (Table 6). In general, substitution of the amino acid residue containing a hydrophobic side chain (phenylalanine, isoleucine, leucine) with alanine residue resulted in peptides with depressed hydrophobicity parameter as compared to that of aurein 1.2. With polar amino acid residues, the effect of the alanine residue substitution on peptide hydrophobicity was much more diverse. Substitution of either the lysine or serine residues resulted in an increase of hydrophobicity parameter. On the other hand, substitution of aspartic acid residue only slightly increased peptide hydrophobicity, while substitution of glutamic acid depressed it. Interestingly, substituting glycine residue with alanine residue also resulted in a peptide with a decreased hydrophobicity parameter. The changes in hydrophobicity are fairly well correlated with amino acid hydrophobicity scale derived from RP-HPLC analyses (Table 6 and Supplementary Fig. 1). However, G1A and F3A, unexpectedly, did not meet these predictions [24].

**Table 6:** Hydrophobicity parameter (%ACN) of the peptides, changes in hydrophobicity (∆%ACN), differences derived from hydrophobicity coefficients of amino acid residues–HC, and self-association tendency parameter (*T*_max_)

Peptide	%ACN	∆%ACN (analog–aurein 1.2)	∆HC (alanine–substituted residue)*	*T*_max_ [°C]
G1A	41.8	− 2.9	− 0.15	30
L2A	38.3	− 6.4	− 3.44	25
F3A	41.1	− 3.6	− 4.74	25
D4A	45.7	+ 1.0	+ 0.26	30
I5A	39.5	− 5.2	− 3.42	20
I6A	38.2	− 6.5	− 3.42	20
K7A	47.9	+ 3.2	+ 1.68	30
K8A	48.0	+ 3.3	+ 1.68	30
I9A	37.8	− 6.9	− 3.42	15
E11A	43.5	− 1.2	+ 0.16	30
S12A	45.7	+ 1.0	+ 0.68	30
F13A	37.8	− 6.9	− 4.74	25
aurein 1.2	44.7	0.0	0.0	30

*Values of hydrophobicity coefficient for the following: Ala (0.06), Asp (− 0.20), Glu (− 0.10), Gly (0.21), Ile (3.48), Leu (3.50), Lys (− 1.62), Phe (4.80), Ser (− 0.62); derived from analyses performed on RP-HPLC system with conditions closely similar to those applied in this study (C18 stationary phase, water/ACN with 0.1% TFA) [24]

### Peptide Tendency to Self-Association

Generally, the tendency to self-association of the peptide was influenced by altered amino acid residue hydrophobicity (Table 6 and Supplementary Fig. 2). Substitution of any of the polar amino acid residues with alanine residue did not result in altered self-association tendency when compared to that of aurein 1.2. On the contrary, in every case of a peptide with non-polar residue (phenylalanine, isoleucine, leucine) substituted with the alanine residue, the self-association tendency was suppressed. This phenomenon was distinct for I9A, in which *T*_max_ was half that compared to *T*_max_ of aurein 1.2 (15 °C and 30 °C, respectively). Importantly, *T*_max_ is not well correlated with %ACN (simple linear regression, *R*^2^ = 0.631). In general, if %ACN of the peptide used in this study was ≤ 41.1%, then *T*_max_ was ≤ 25 °C; and if %ACN was > 41.1%, then *T*_max_ was 30 °C (Supplementary Fig. 2).

### Serum Stability of Peptides

Serum stability of the peptides was expressed as percentage of molecule which remained after 6-h incubation in FBS solution at 37 °C (Table 7). Almost all peptides, with the exception of S12A and I5A, were less stable than aurein 1.2. The most stable molecule, S12A, retained 77% of initial concentration, while the least stable molecules, L2A and I9A, were degraded down to 6% of initial peptide concentration.

**Table 7:** Percentage of peptide remaining after 6 h of incubation at 37 °C in serum

Peptide	Remaining peptide [%]
G1A	16
L2A	6
F3A	38
D4A	44
I5A	71
I6A	29
K7A	59
K8A	36
I9A	6
E11A	17
S12A	77
F13A	54
aurein 1.2	65

### CD Results

**Fig. 1:**
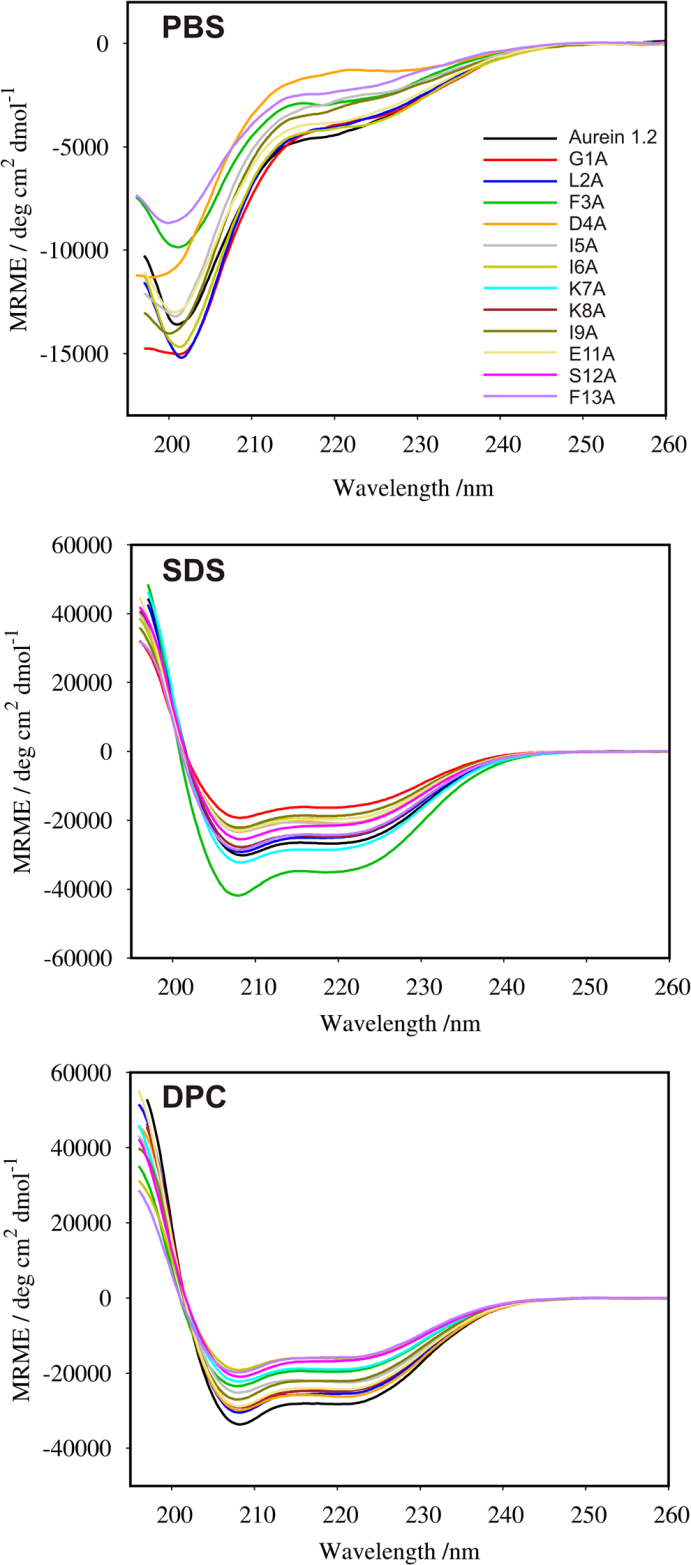
Far-CD spectra of aurein 1.2 and its alanine scanning analogs recorded in a surfactant-free phosphate buffer (PBS), buffered sodium dodecyl sulfate (SDS), and dodecylphosphocholine (DPC) solutions

CD spectra of aurein 1.2 and its alanine scanning analogs are shown in Fig. 1. Due to poor solubility, the CD spectra of three analogs (K7A, K8A, and S12A) could not be recorded in surfactant-free phosphate buffer solution. The spectra of the remaining peptides showed a minimum around 200 nm revealing the state of random conformation. Addition of surfactants, SDS or DPC, induced formation of a helical structure with two characteristic minima at 208 and 222 nm in all the CD spectra. This supports the hypothesis that the peptides adopt helical structure upon interaction with biological membranes as expected for this class of antimicrobial compounds. The *content* of helical structure predicted from the *CD* spectra is displayed in the Table 8. As seen, the replacement of originally occurring amino acids with alanine residue reduced helical structure content, except for two analogs, F3A and K7A, in SDS solution, where a slight increase in helicity is seen. Interestingly, Θ222/Θ208 ratio of all peptides in our study was lesser or equal to 0.9. Therefore, regardless of used surfactant, a coiled coil structure was adopted by none of studied peptides.

**Table 8:** α-Helical content and the Θ222/Θ208 ratio determined by CD. Deconvolution of the CD spectra was carried out using CDPro software with CONTINILL algorithm

Peptide	SDS		DPC	
	Helicity %	Θ222/Θ208	Helicity %	Θ222/Θ208
G1A	63	0.82	91	0.81
L2A	84	0.84	89	0.83
F3A	94	0.82	69	0.83
D4A	71	0.88	87	0.87
I5A	72	0.90	79	0.89
I6A	70	0.86	67	0.84
K7A	89	0.86	78	0.85
K8A	76	0.87	89	0.85
I9A	68	0.83	80	0.81
E11A	75	0.84	87	0.77
S12A	76	0.83	74	0.79
F13A	72	0.83	61	0.80
aurein 1.2	86	0.87	95	0.84

## Discussion

Because of their wide-spectrum activity and low-resistance development propensity, AMPs are perceived as potential candidates for anti-infective drugs. Despite this, none of the studied molecules has completed the clinical trials with a positive outcome thus far. As an example, pexiganan (MSI-78), a 22-residue magainin analog, formulated as a 0.8% cream (Locilex) have recently failed to pass Phase III clinical trials. Locilex did not exhibit any superiority against a placebo cream in treating mild infections of diabetic foot ulcers [25]. There are several reasons for limitation of marketing AMPs, such as high production cost, enzymatic and chemical degradation vulnerability, and challenging formulation development, to name few of them.

Aurein 1.2 is one of the shortest nature–derived AMPs [26]. Owing to only 13 amino acid residues in its backbone and a fair antimicrobial activity, aurein 1.2 appears to be an interesting anti-infective drug candidate. In addition, further modifications of its amino acid residues might provide an analog with an increased biological activity and chemical stability.

Up to date, a number of studies describing structure-activity relationship of aurein 1.2 have been published. Li et al. found Phe13 residue to be an essential asset for antimicrobial activity. In this study, aurein 1.2 and its two analogs F13W (Phe13 residue substituted with Trp) and F13X (Phe13 residue substituted with Phg) were compared regarding their helicity, hydrophobicity, and antimicrobial activity against *E. coli*. Both substitutions led to peptides with a lesser antimicrobial activity than that of aurein 1.2. As the studied molecules were characterized by similar helicities, it was suggested that this decrease in activity resulted from a different location of aromatic ring of the last residue relative to peptide backbone [27]. This suggestion was further explored by Shahmiri et al.. According to the authors, both phenylalanine residues of aurein 1.2 play a crucial role in membrane anchoring, with Phe13 being more influential [28]. Again, Shahmiri et al. recognized that aurein 1.2 C-terminal amidation directly influences membrane disruption through promotion of peptide aggregation [29]. Soufian et al. reported increase in antimicrobial activity of aurein 1.2 analog in which N-terminal glycine was substituted with arginine. They suggest that this augmentation of antimicrobial activity is the result of a higher net charge [30]. Furthermore, Sajjadiyan et al. assigned an essential role in lytic activity of aurein 1.2 to Phe3, Lys7, and Lys8. They suggest that those three amino acid residues play a crucial role in absorbing water molecules into the membrane [31].

**Fig. 2:**
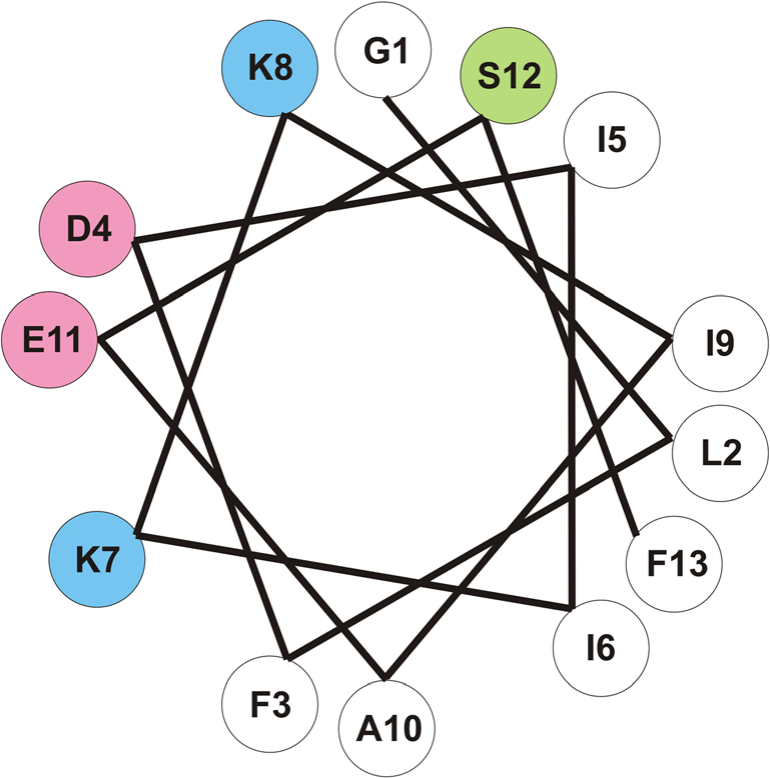
Helical wheel projection of aurein 1.2. Pink color represents acidic amino acids, blue–basic amino acids, green–polar, and uncharged amino acid and blank–hydrophobic amino acids

In our study, alanine scanning was conducted to establish a relationship among the structure, antimicrobial activity, and stability of aurein 1.2. As a result, several interesting observations were made. Substitution of Asp4 and Glu11 residues resulted in molecules with enhanced antimicrobial activity against almost all strains of the microorganisms. Those negatively charged residues make up the central part of the hydrophilic face of aurein’s 1.2 α-helix (Fig. 2). Thus, the mutation of each of them into an alanine residue affected the amphipathicity of the helical structure and, consequently, produced analogs with a depressed helicity. Interestingly, this outcome did not affect the antimicrobial activity in a negative sense. Moreover, as no significant difference in *T*_max_ parameter between E11A, D4A, and aurein 1.2 has been found, the increased antimicrobial activity could also not be related to an enhanced tendency to self-association. In addition, both analogs exhibited a lower proteolytic stability than their counterpart (44%, 17%, and 65% of remaining peptide after 6-h incubation in bovine serum, respectively) suggesting that the enhanced antimicrobial activity could not be the result of extended time of action. The most reliable reason for this enhancement of the activity is an increased positive net charge of the analogs. Placing electrically neutral alanine residue in position of aspartate and glutamate residues resulted in a peptide with a negatively charged microbial membrane interaction enhancement and elimination of electrostatic repulsion.

All analogs with hydrophobic residue (phenylalanine, isoleucine, or leucine) substituted with the alanine residue were characterized by a lower antimicrobial activity than that of aurein 1.2. This phenomenon might be the result of cumulative influence of the reduced *T*_max_ parameter, hydrophobicity, and helicity of the analogs. Substitution of hydrophobic side chains on the non-polar face of the aurein 1.2 α-helix with a shorter side chain of alanine is likely to affect hydrophobic interactions essential for the peptide mechanism of action. Moreover, it might reduce self-association tendencies, helical structure stabilization, and anchoring of the peptides into the microbial bilayers, this ultimately leading to suppression of the antimicrobial activity. The importance of hydrophobic side chains in self-association and irrelevance of non-hydrophobic residues strongly suggest that peptide-peptide attraction relies on interactions between hydrophobic faces of aurein 1.2. It has been found that hydrophobicity (retention time) of α-helical peptides is correlated with peptide self-association ability, helicity, hemolysis, and antimicrobial activity. Moreover, antimicrobial activity versus hydrophobicity has an optimum window. If it is exceeded, antimicrobial activity will decrease [32]. Supplementary Figs. 3–7 present the change in hydrophobicity of the peptides (%ACN _of analog_ − %ACN _of aurein 1.2_) versus change in MIC (log_2_*X*, where *X* is a quotient of the MIC of an analog by the MIC of aurein 1.2). Importantly, a value of 1024 was applied for calculations when MIC was > 512 μg/mL. With *S. aureus*, MIC apparently depends on the peptide hydrophobicity and the net charge. K7A and K8A are more hydrophobic than aurein 1.2, but their net charge is lower. The effect of reduced net charge overweighs the increased hydrophobicity, this leading to a reduced antimicrobial activity of these analogs. The increased net charge also overweighs effect of reduced hydrophobicity. As a result, E11A has a higher antistaphylococcal activity than the parent molecule. The strongest antimicrobial analog (D4A) has both the higher hydrophobicity and net charge than aurein 1.2. Moreover, substitution of Ser12 also resulted in an analog with a higher hydrophobicity and antistaphylococcal activity. With other microorganisms, S12A was equally active or a less active against *P. aeruginosa* than the parent molecule. Similar to D4A and E11A, S12A was characterized by an equal *T*_max_ parameter as compared to that of aurein 1.2. However, in contrast to those analogs, the net charge of S12A equals to that of aurein 1.2. Taking into account the fact that S12A retained 77% of initial concentration after 6 h incubation in bovine serum solution (37 °C), the increased antimicrobial activity, as compared to that of aurein 1.2, could be linked with its increased stability. However, as bovine serum might significantly differ from microbial culture medium regarding enzyme composition, further comparative studies are necessary. Moreover, it seems that the serine residue interacts with bacterial membrane components, this being vital for membrane disruption. This effect is irrelevant with *S. aureus*, but essential for *P. aeruginosa* cell surface-peptide interaction (Supplementary Fig. 6). Surprisingly, although both K7A and K8A peptides were characterized by identical nearest-neighbor effect of substituted lysine residue, net charge, *T*_max_ values, and similar hydrophobicities (%ACN), they exhibited considerable differences in biological activity. While K8A showed depressed antimicrobial and increased hemolytic activities as those of aurein 1.2, K7A was almost completely depleted of biological activity. Those differences can be due to unequal α–helical contents in the SDS and DPC micelles. Substitution of Lys8 residue with alanine led to a molecule with a higher α–helical content in DPC micelles (mimicking mammalian membranes) and lower in SDS micelles (mimicking bacterial membranes) as compared to that of the K7A analog. In general, K7A adopts a more helical structure in SDS (89%) than in DPC (78%), and helicity of K8A is higher in DPC (89%) than in SDS (76%). Furthermore, only the helicity of K7A in SDS is higher than that of aurein 1.2 (86%). Hypothetically, if the helicity is a critical factor for antimicrobial activity, and the hydrophobicity remains similar, it should be found that K7A has a stronger antimicrobial activity and lower hemolysis than K8A. However, antimicrobial activity of K8A against *E. faecalis*, *E. coli*, and *C. albicans* is markedly higher (Supplementary Figs. 4, 5, and 7, respectively). Interestingly, increasing the net charge (+ 2) and/or hydrophobicity of aurein 1.2 analogs does not result in a stronger anticandidal activity. However, reducing the net charge has a negative effect (Supplementary Fig. 7). Presumably, Asp4, Glu11, and Ser12 may contribute to in peptide-yeast cell interaction and this factor overweighs the impact of the increasing hydrophobicity and net charge of respective analogs. An opposite effect of substitution of negatively charged residues is evident in the case of eradication of *E. faecalis* biofilm—D4A has distinctly lower MBEC than E11A (Table 3). Interestingly, S12A has a similar activity to that of D4A which definitely surpasses the parent molecule in this regard. Those findings indicate that Glu11 can contribute to disruption of the mature *E. faecalis* biofilm. However, it is worth noting that this analog is at the same time more hydrophilic than aurein 1.2, this could partially diminish the effect of the increasing net charge. Scanning of the aurein 1.2 peptide library revealed similar tendencies of the most of the microbial strains in microbiological assays (i.e., D4A, E11A, and S12A were characterized by enhanced activity, whereas activities of the remaining peptides were similar or decreased when compared to aurein 1.2). Nevertheless, a deviation from this trend was noticed in the case of *C. albicans* and the measurements of adhesive properties. Furthermore, eradication of mature structures was observed only for aurein 1.2 and three other analogs. On the other hand, all the tested compounds were found to inhibit the early stages of *Candida* biofilm formation. This can be explained by the fact that aurein 1.2 and its analogs can interfere with the expression of genes involved in the induction of hyphae formation, adhesion, and extracellular matrix production such as Ras1-cAMP-Efg1 and MAP kinase [33–35]. However, this statement should be supported by appropriate analyses of gene expression. Another hypothesis claims that the test compounds could bind to carbohydrates making up part of *C. albicans* cell wall and which particularly take part in adhesion [36]. However, Lorenzón et al. in their study on aurein 1.2 and mannan interactions do not support the hypothesis that aurein 1.2 interacts with the main carbohydrate of the yeast cell wall [37]. On the other hand, the dimeric analog of aurein 1.2 effectively binds to mannan and aggregate *C. albicans* [13]. Peptide analogs used in this study were ineffective against yeast biofilm. Only the parent molecule and S12A exhibited noticeable antibiofilm activity.

Hemolysis of the analogs at 256 μg/mL is shown in Supplementary Fig. 8. It is noteworthy that all the peptides that are more hydrophilic than aurein 1.2 are simultaneously less hemolytic. Furthermore, analogs that differ from the parent molecule in hydrophobicity by more than − 3.6 %ACN evoke little or no hemolysis. Five peptides were included in a simple linear regression to visualize hypothetical curve of hemolysis vs hydrophobicity (G1A, F3A, K8A, aurein 1.2, and E11A; *R*^2^ = 0.967), but compounds of this group carry different net charges. Importantly, D4A, K7A, and S12A are outside of the hypothesized linearity. In fact, D4A and S12A similar to K7A exhibited a lower helicity in DPC than did aurein 1.2 (87% and 74%, respectively). Probably, Asp4 and Ser12 interact with RBC membrane components like zwitterionic phospholipidic headgroups and thus substitution with alanine leads to lower hemolysis. Both lysine residues have identical nearest neighbors, isoleucine, and lysine, and thus the difference in K7A and K8A biological activity results presumably from interactions with more distant residues. In fact, aurein 1.2 is capable to form five salt bridges between N-terminal amino group or side chain of the lysine residue and side chain of aspartic or glutamic acid (distance *i + i + 3/i + 4*; Table 9). With E11A, the measured change in hydrophobicity (the analog is more hydrophilic) is in contrast to predictions derived from hydrophobicity coefficients (Table 6, Supplementary Fig. 1). This may be due to the phenomenon of charge compensation between the carboxyl and amine groups, i.e., zwitterionic alpha amino acids are more hydrophobic than could be predicted from the occurrence of two charged moieties [38]. Cheraghi et al. in molecular dynamics study of aurein 1.2 (5-peptide channel, with hydrophilic sides of peptides directed towards the center of pentagon channel) have shown that only in one case (in one chain, Lys8-Asp4) the residues are far enough to be able to neutralize each other [39]. Furthermore, it is possible that Asp4 is neutralized with the N-terminal amino group (*i*, *i + 3*); hence, Asp4 can interact less effectively with Lys7 and Lys8 (*i*,*i + 3* and *i + 4*, respectively). The previous studies on amphipathic α-helical cationic antimicrobial peptides indicate that an increase in net charge and hydrophobicity leads to stronger antimicrobial activity and hemolysis, but when the positively charged amino acid is located on the non-polar face it can reduce toxic effect [40]. Obviously, Lys8 is not located on the non-polar face of aurein 1.2 (Fig. 2); nevertheless, the effect of its substitution with alanine resulted in a compound with the highest hemolytic activity. Substitution of Lys7 residue (in the vicinity of peptide polar/non-polar face) resulted in a peptide with a markedly reduced biological activity. In conclusion, K8A is more hemolytic due to enhanced hydrophobicity and K7A is less hemolytic due to the reduction of the net charge that outweighs the influence of enhanced hydrophobicity. In other words, effective charge of Lys7 predominates effective charge of Lys8 due to compensation effect. Further considerations lead to the following hypothesis: the side chain of Glu11 partially compensates positive charge of the Lys8 (*i*,*i + 3*) side chain and the N-terminal amino group partially compensates Asp4. In effect, Lys7 interacts more efficiently with biological membranes than does Lys8. Our study revealed that substitution of Lys7 and Lys8 with alanine resulted in identical antimicrobial activities against *S. aureus* and *P. aeruginosa* (over the range of 1–512 μg/mL), as opposed to other microbial strains and RBCs. Nonetheless, previous studies on aurein 1.2 have shown that hydrophobic interactions play a key role in helicity of peptide bound to micelles and salt bridges are irrelevant and their existence is uncertain [41].

**Table 9:** Distances of salt bridges between N-terminal amino group or side chain of lysine residues and side chain of aspartic or glutamic acid

	Asp4	Glu11
N-terminus	*i,i + 3*	Irrelevant
Lys7	*i,i + 3*	*i*,*i + 4*
Lys8	*i*,*i + 4*	*i,i + 3*

Individual hydrophobicity of alanine residue is higher than that of glycine. Nevertheless, substitution of the former residue with alanine, unexpectedly led to G1A—an analog characterized by a lesser hydrophobicity than that of aurein 1.2. In addition, a significant difference between helical content in SDS and DPC micelles was noticed. α-Helical content of G1A was by ca. 25% smaller in SDS micelles than in DPC. Bearing in mind that SDS micelles, as well as bacterial membranes, are negatively charged, it suggests an essential role played by glycine residue in microbial selectivity. However, as a decrease was found in both antimicrobial and hemolytic activities, evidently, glycine residue in the case of aurein 1.2 contributes to membrane interaction in general.

In summary, helicity, hydrophobicity, and aggregation tendencies play an essential role in microbial specificity of AMPs. Generally, increase in those values to definite extent results in enhancement of antimicrobial activity. Further augmentation, however, not only reduces antimicrobial activity due to peptide aggregation in solution, but also strengthens hemolytic activity of the molecule [32, 42, 43]. Similar tendencies emerged in the case of studied alanine scan analogs. Aurein 1.2 is characterized by the highest helicity, *T*_max_, parameter, and significant hydrophobicity, at the same time exhibited one of the highest hemolytic activities among the components of the peptide library. While observations made for D4A were comparable to those of aurein 1.2, substitution of glutamic acid residue with alanine resulted in a molecule (E11A) with a slightly lower hydrophobicity and, in consequence, in hemolytic activity. On the other hand, a considerable decrease in α-helical content, as seen in S12A, also resulted in reduction of hemolysis, while retaining the antimicrobial activity. Along with their antimicrobial activity, all analogs with hydrophobic amino acid residues (phenylalanine, isoleucine, or leucine) substituted with alanine exhibited a huge drop in hemolytic activity, again, as a result of the decrease in hydrophobicity, *T*_max_, and helicity.

An article with part of experimental section titled “Site-directed mutagenesis of aurein 1.2” has been published so far [44]. However, in fact, the object of alanine scan described there is no aurein 1.2 peptide itself, but a conjugate of aurein 1.2 with + 36 GFP-Cre recombinase fusion protein. Even so, our study is the first one in which the influence of each amino acid residue substitution on aurein 1.2 antimicrobial, antibiofilm, and hemolytic activities, secondary structure, hydrophobicity, aggregation tendencies, and serum stability have been comprehensively characterized. The correlations established between those characteristics led to selection of key residues essential for peptide activity. Moreover, residues which did not affect at all, or even negatively affected biological activity of aurein 1.2, were also appointed. Those outcomes can assist in aurein 1.2-based molecule design optimization. For instance, peptides with increased antimicrobial activity may be obtained by substitution of both Asp4 and Glu11 with neutral or positively charged residues. On the other hand, studies on substitution of Ser12 might result in antimicrobial compounds with selectivity against *S. aureus*. Additionally, other modifications such as dimerization [45], hydrocarbon stapling [46], or lipidation [47] can be also applied to improve pharmacological properties of aurein 1.2.

## Electronic Supplementary Material

ESM 1 (PDF 236 kb)
