# Supplementary material for: Alanine Scanning Studies of the Antimicrobial Peptide Aurein 1.2
Source: Probiotics Antimicrob Proteins. 2018 Dec 19;11(3):1042–54. doi: 10.1007/s12602-018-9501-0 (PMC6695355; doi:10.1007/s12602-018-9501-0)
Supplement: Supplementary file 1 — (PDF 236 kb) [file 12602_2018_9501_MOESM1_ESM.pdf]

ALANINE SCANNING STUDIES OF THE ANTIMICROBIAL PEPTIDE AUREIN 1.2
SUPPLEMENTARY MATERIAL

DORIAN MIGOŃ[1, 2], MACIEJ JAŚKIEWICZ[1], DAMIAN NEUBAUER[1], MARTA BAUER[1], EMILIA SIKORSKA[3], ELŻBIETA KAMYSZ[3], WOJCIECH KAMYSZ[1]

[1]*Department of Inorganic Chemistry, Faculty of Pharmacy, Medical University of Gdańsk, Gdańsk, Poland*

[2]*Polpharma Biologics, Gdańsk, Poland*

[3]*Faculty of Chemistry, University of Gdańsk, Poland*

Abstract: Antimicrobial peptides (AMPs) are compounds widely distributed in nature that display activity against a broad spectrum of pathogens. Amphibian skin, as an organ rich in pharmacologically active peptides, appears to be an interesting source of novel AMPs. Aurein 1.2 (GLFDIIKKIAESF-NH_2) is a short 13-residue antimicrobial peptide primarily isolated from the skin secretions of Australian bell frogs. In this study, the alanine scan of aurein 1.2 was performed to investigate the effect of each amino acid residue on its biological and physico-chemical properties. The biological studies included determination of minimum inhibitory concentration, activity against biofilm and inhibitory effect on its formation. Moreover, the hemolytic activity as well as serum stability were determined. The hydrophobicity of peptides and their self-assembly were investigated using reversed-phase chromatography. In addition, their helicity was calculated from circular dichroism spectra. The results not only provided information on structure-activity relationship of aurein 1.2 but also gave insights into design of novel analogs of AMPs in the future.

Keywords: aurein 1.2, antimicrobial peptides, structure-activity relationship, antimicrobial agents, peptide drugs, antibiotics

*Address correspondence to this author at the Department of Inorganic Chemistry, Faculty of Pharmacy, Medical University of Gdańsk, Al. Gen. J. Hallera 107, 80-416, Gdańsk, Poland; Tel/Fax: +48-58-349-14-88; e-mail: dorianmig@gumed.edu.pl

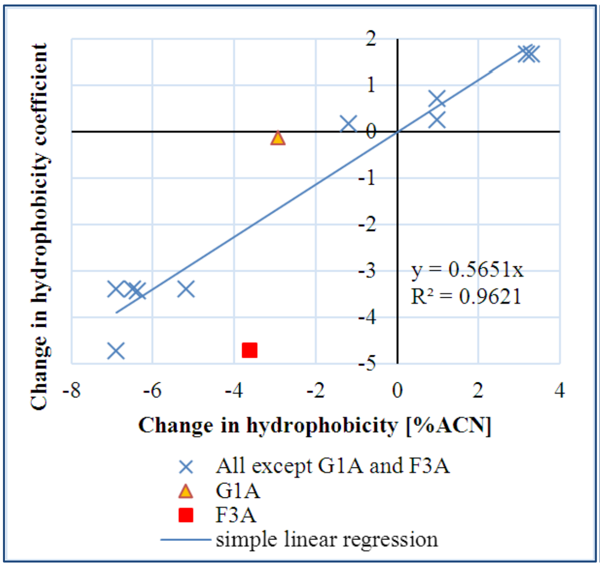

Figure 1 Change in hydrophobicity [%ACN] *vs* change in hydrophobicity coefficient.

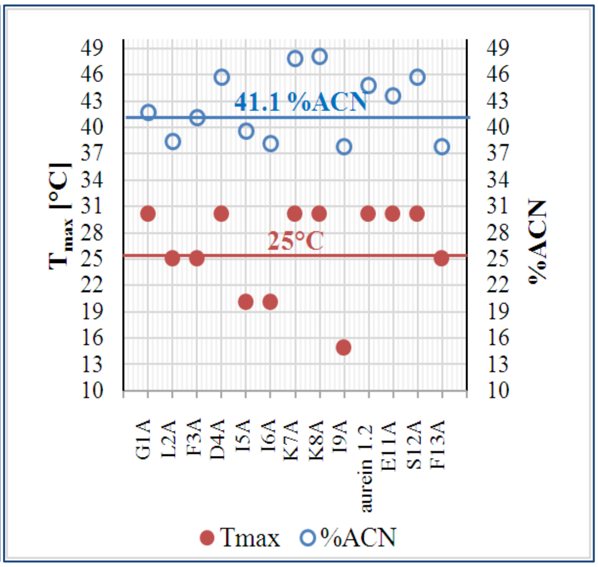

Figure 2 A graphic presentation of T_{max} and %ACN.

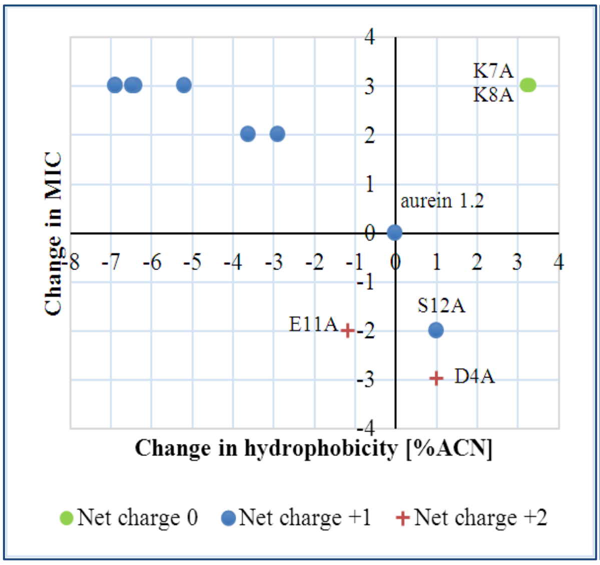

Figure 3 Change in hydrophobicity *vs* change in antimicrobial activity against *S. aureus*.

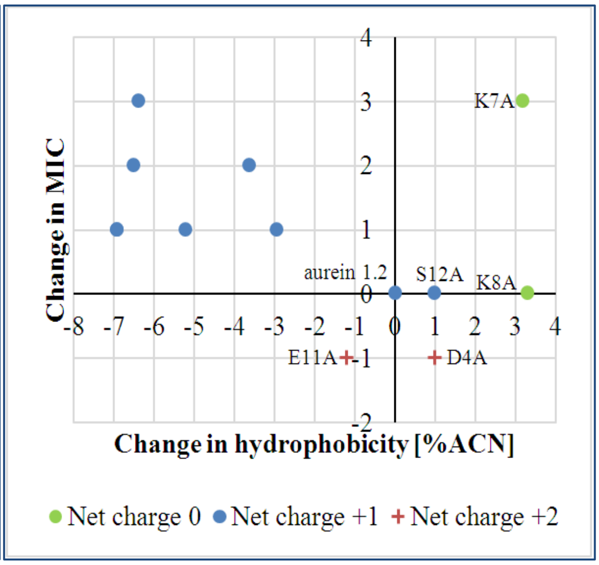

Figure 4 Change in hydrophobicity *vs* change in antimicrobial activity against *E. faecalis*.

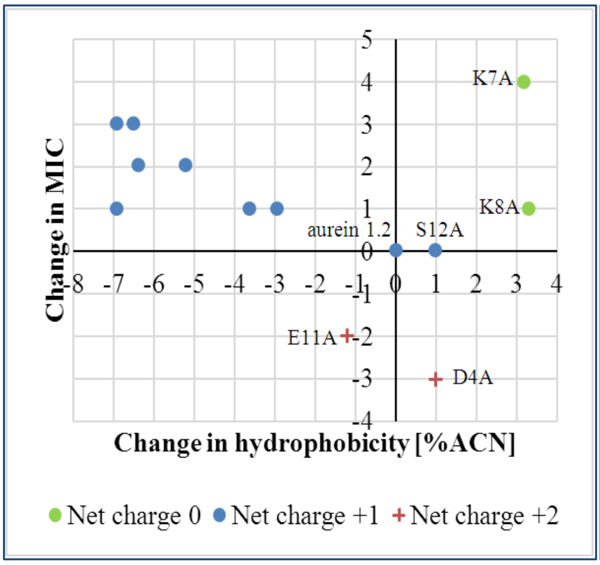

Figure 5 Change in hydrophobicity *vs* change in antimicrobial activity against *E. coli*.

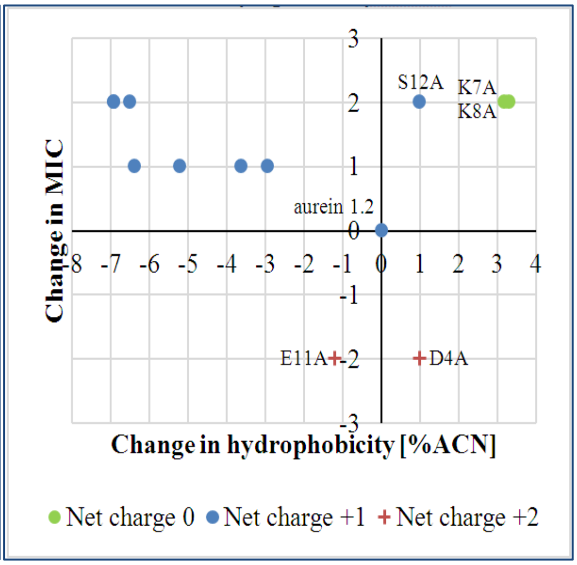

Figure 6 Change in hydrophobicity *vs* change in antimicrobial activity against *P. aeruginosa*.

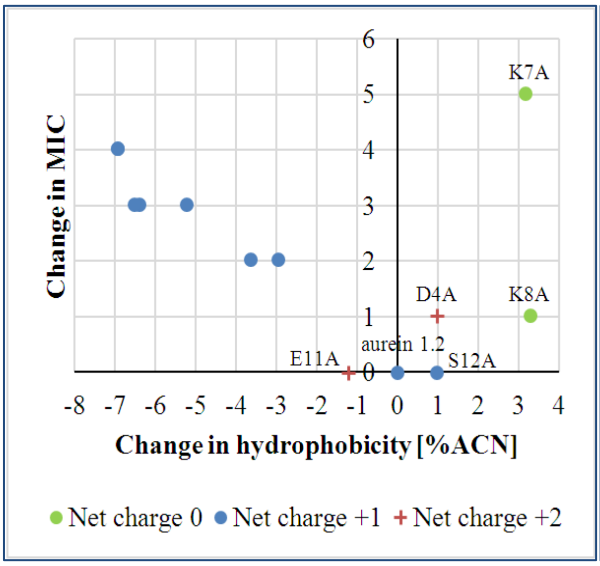

Figure 7 Change in hydrophobicity *vs* change in antimicrobial activity against *C. albicans*.

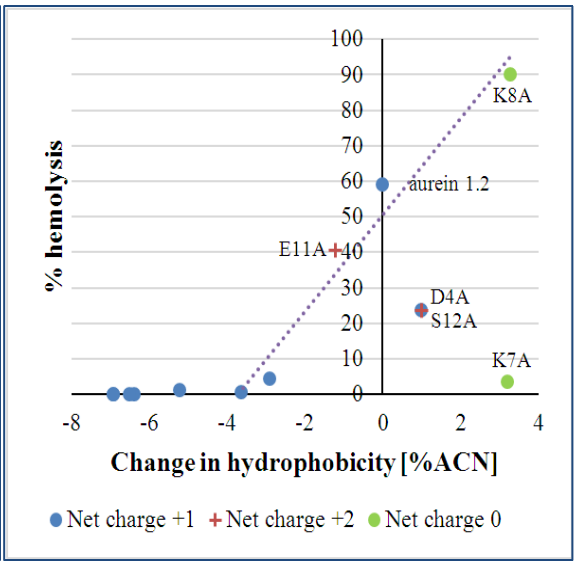

Figure 8 Change in hydrophobicity *vs* % hemolysis at 256 µg/mL.
